# Patient-Reported Outcomes of Bladder and Bowel Control in Children with Spina Bifida

**DOI:** 10.3390/children8030209

**Published:** 2021-03-10

**Authors:** Helen Träff, Anna Börjesson, Martin Salö

**Affiliations:** Department of Clinical Sciences, Pediatrics, Lund University, 22185 Lund, Sweden; helen.traff@med.lu.se (H.T.); anna.borjesson@skane.se (A.B.)

**Keywords:** bowel management, neurogenic bladder, outcome, spina bifida, surgery

## Abstract

Background: The primary aim was to describe patient-reported morbidity from neurogenic bladder and bowel dysfunction in a cohort of children with spina bifida. The secondary aim was to describe the overall surgical burden in these children. Methods: Children with meningocele or myelomeningocele, born between 2000–2016, and followed by a tertiary spina bifida center were evaluated in a cross-sectional cohort study using data from charts and a prospective national follow-up program. Results: In the group of 62 patients, clean intermittent catheterization (CIC) was used by 47 (76%) of the patients, and anticholinergic treatment was used by 36 (58%). More than one third of the patients reported inadequate results with daily urinary leakage. Laxatives and enema were used regularly by 45 (73%) and 39 (63%) patients, respectively. Inadequate results were reported by seven (11%) patients. One or more urogenital or gastrointestinal operations had been performed in 26 (42%) patients, with a total of 109 procedures overall. Conclusions: Despite substantial bowel and bladder management, a significant portion of children suffered from inadequate results concerning bladder and bowel control. Many surgeries were performed in a defined group of the children. Prospective, long-term studies can evaluate if more aggressive medical and/or surgical management could increase bowel and bladder control.

## 1. Introduction

Around 15–20 children in Sweden, and 400,000 worldwide, are born with spina bifida each year [1]. Most of these children suffer from neurogenic bladder and bowel dysfunction which may result in severe constipation, urinary and fecal incontinence, vesicoureteral reflux, urinary tract infections, and high intravesical pressures with risk of renal damage [2,3]. Improved management of children with spina bifida has led to a higher rate surviving to adulthood, although a high mortality risk exists throughout adult life [4,5]. During childhood, these children are subjected to extensive medical investigations and interventions [6]. However, it is not clear whether these result in a true increase in quality of life for these patients and their caregivers [4].

Based on a national follow-up program, an evaluation of the current treatment situation for neuropathic bladder and bowel dysfunction in children with spina bifida in different regions of Sweden was published locally in 2017. In conclusion, great regional differences were seen regarding the age of starting clean intermittent catherization (CIC), the use of anticholinergic treatment and enemas, and the rate of surgical procedures for incontinence. Sweden’s southern healthcare region, the area investigated in this study, showed a restrictive picture throughout all of these parameters, with a low percentage of pharmaceutically and surgically treated patients [7].

The primary aim of this study was to describe the patient-reported morbidity from neurogenic bladder and bowel dysfunction in children with spina bifida, and the secondary aim was to shed light on their overall surgical burden during childhood, with a focus on urogenital and gastrointestinal operations. The collected information is of value to both healthcare professionals and families by increasing knowledge regarding the management and outcome of urinary tract and gastrointestinal function during childhood and adolescence in children with spina bifida.

## 2. Materials and Methods

### 2.1. Settings and Patients

The original data set included all children born from 2000 to 2016, diagnosed with a disease or defect of the spinal cord, and monitored by the spina bifida center in Sweden’s southern healthcare region. The center covers an area of around 1.8 million inhabitants. Only patients diagnosed with meningocele or myelomeningocele were included in the study. Patients who had other disorders of the spine, who were lost before follow-up, or who were deceased were excluded.

### 2.2. Study Design

This was a retrospective, cross-sectional cohort study of data collected from a prospective register. Data for patients was gathered from the date of birth until 31 August 2017. In addition to basic information about the patient group, such as age, gender, and details regarding the diagnosis, concomitant congenital malformations were identified. For the urogenital and gastrointestinal spectrum, variations in treatment methods and the quantities as well as types of surgical interventions were calculated.

### 2.3. Definitions

To determine if patients suffered from meningocele or myelomeningocele, operation charts, MR imaging, and pathology samples were carefully studied. Treatments were defined using medical records from the last follow-up. Regarding patient-reported outcomes of bowel and bladder management, three types of answers were used: satisfying, acceptable, and inadequate and were assessed by two of the authors. Satisfying corresponded to leakage or other problems, maximum once/month; acceptable corresponded to problems every week; and inadequate corresponded to daily problems with significant leakage. Because of the retrospective design, it was impossible to use standardized evaluation forms. Patients were placed in respective groups depending on reports from the last follow-up. All specific surgical procedures, even those being performed during the same session, were counted as individual procedures.

### 2.4. Statistical Analysis

A Mann–Whitney U-test was used to compare continuous non-parametric data. To compare categorical data between more than two groups, a chi-square test with a post hoc test (Bonferroni) was used. SPSS (Statistical Package for Social Sciences), version 24, was used for the calculations. Statistical significance was set to *p* < 0.05.

## 3. Results

From the original data set of 84 patients, 62 children were included in the final data set after excluding patients with other disorders of the spine (*n* = 17) and patients lost to follow-up (*n* = 3) or deceased (*n* = 2). The study group had an equal gender distribution with 32 (52%) female and 30 (48%) male patients (Table 1). Of the included patients, 47 (76%) had a myelomeningocele (MMC) and 5 (8%) had a meningocele (MC). A specific diagnosis of MC or MMC could not be defined in 10 (16%) of the patients since they underwent neurosurgical closure of their spina bifida in another country. A ventriculoperitoneal shunt had been placed in 28 (45%) of the patients, and 32 (52%) had a Chiari malformation in the brain. A wheelchair was used by 25 (40%) of the patients. In total, 28 (45%) of the patients had one or more malformations within the gastrointestinal or urogenital tract, with vesicoureteral reflux (VUR) being the most common and found in 21 (34%) patients (Table 1).

At the latest follow-up, clean intermittent catheterization was used by 47 (76%) patients. Anticholinergic treatment for neurogenic bladder dysfunction was used by more than half of the patients (58%). There were eight (13%) patients who had undergone 18 surgical interventions related to urinary incontinence, detrusor Botox injection, bladder neck injection with a bulking agent, or bladder augmentation with an alternative catheterizable channel.

Half of the patients described their present management of urinary incontinence as satisfying (35%) or acceptable (26%), while more than one-third suffered from inadequate results with daily leakage. Of these patients, 17 (74%) were using both CIC and anticholinergic treatment, and two (9%) patients were also treated with bladder neck injection. The remaining patients only used CIC (13%) or did not use CIC or anticholinergic treatment (13%). When comparing the type of spina bifida, the presence of CIC, or the rate of treatments (medical or surgical) between the different groups of self-reported outcome, no significant differences were found (data not shown). At the latest follow-up, three patients (5%) had a glomerular filtration rate based on cystatin C (Pt-eGFR (CyC)) lower than the reference; two of these had affected kidney function confirmed by iohexol clearance and radiologic findings (Table 2).

Voiding cystourethrogram (VCUG) was only available in 55 patients. Vesicoureteral reflux was evident in 21 (34%) patients at the first VCUG. Of these, 15 patients had VUR of grade 3 or higher and hence, fulfilled the indication for treatment with prophylactic antibiotics and follow-up with a second VCUG. A second VCUG was performed in 13 patients, in which nine (69%) patients still had VUR of grade 3 or higher. Of these, three (34%) patients underwent surgical treatment for reflux (Table 3).

Use of laxatives and enema was common with 45 (73%) and 39 (63%) of the patients treated regularly with each of these treatments, respectively. Appendicostomy for Malone antegrade colonic enema (MACE) had been performed in four (6%) patients. Just above half of the group reported a satisfying result regarding their bowel management at the latest follow-up. An acceptable result was described by 22 (35%) patients, who experienced problems with either constipation or soiling (Table 4). Inadequate results were reported by seven (11%) patients, who experienced problematic constipation or daily fecal leakage. From this group, six (86%) patients used laxatives and enemas regularly but had no gastrointestinal surgery for their problems. In the four patients that underwent appendicostomy surgery for antegrade enemas (all of them with MMC), one had a satisfying result, and three had acceptable results. In summary, when comparing the type of spina bifida, the rate of treatments (laxatives, enema), or the rate of appendicostomy operation between the different groups of self-reported outcomes, children in the satisfied group were less likely to be treated with enema (*p* < 0.001) but no other significant differences were found.

Among the 62 patients, 26 (42%) underwent one or more surgeries due to urogenital or gastrointestinal conditions related to spina bifida or other congenital malformations (Table 5). The total number of surgeries was 109, with a median of 3 (1–13) surgeries/patient. Overall, 18 (29%) patients underwent a total of 58 urogenital surgeries, with a great variety in complexity. Gastrointestinal surgery was performed in 14 (23%) patients, with inguinal hernia repair being the most common. When comparing the rate of surgery among different groups with spina bifida, more children with MMC had undergone one or more surgeries (51%) compared to children with MC (20%) or children in the unknown group (10%) (*p* = 0.03).

## 4. Discussion

In this cross-sectional, retrospective study, a significant portion of the children reported inadequate results concerning bladder and bowel control despite substantial bladder and bowel management. A high surgical burden was found to be concentrated within a defined group of children with MMC.

The percentage of children using CIC was equal with that in the study of children with MMC born in Sweden between 1986 and1989 (83% vs. 85%) [8] but higher than international studies of children with spina bifida [9,10]. Anticholinergic drugs were used to a greater extent in this study (68%) than in both national and international studies (40–41%) [8,9]. One reason might be that there exists a greater knowledge regarding the benefits of early anticholinergic treatment now than previously, or this may indicate a more aggressive approach to pharmaceutical treatment in Sweden’s southern healthcare region. It could also indicate that a higher number of surgical procedures to relieve bladder pressure are performed in other regions. In our study, only two patients were surgically treated with bladder augmentation, possibly demonstrating a restrictive approach to continence surgery in this patient group. Implementing a standardized, validated patient-reported outcome score in the follow-up of patients with spina bifida might also affect treatment and hopefully, lead to a better patient-reported outcome as compared to the numbers we found in this study [11,12].

According to the Swedish national guidelines for postnatal follow-up of children with spina bifida, the first VCUG examination should be performed within the first 30 days of neonatal life. The results of the present study, with a median age of 29 days at examination among the patients born in Sweden, indicates adherence to this protocol. Unsurprisingly, a higher median age was found in the patient group born in another country, due to the delayed medical investigation in these children. Seven patients were never examined with a VCUG after inclusion to our national follow-up program and therefore, their reflux grade has remained unknown. Six of these patients were born elsewhere, clearly showing the inadequacy of follow-up in newly arrived children. Furthermore, there were also a number of patients with reflux who did not receive antibiotic prophylaxis or their follow-up VCUG according to the national follow-up program for VUR. This information raises questions regarding lack of knowledge among healthcare professionals concerning the guidelines for spina bifida in children and highlights the importance of observing established routines in children with spina bifida. Clear guidelines concerning treatment and follow-up for VUR in patients with neurogenic bladders would be helpful in this patient group.

Bowel management is important in children with spina bifida, and regular treatment with transrectal irrigation has been shown to improve the continence level and minimize constipation in these patients by increasing anorectal sensation and perception [13]. This treatment regimen does, however, also have a practical and social downside, as it is time-consuming as well as poses a problem for reaching independency. Nevertheless, fecal incontinence of the child is often a stressful factor, affecting the quality of life [14,15]. An easier, but not always sufficient, method is the use of oral laxatives, which 73% of the patients in this study used regularly. When comparing this to previous studies [9,10,15,16], this is a higher amount, showing how this treatment constitutes the base of bowel management in patients in Sweden’s southern healthcare region. For many patients, however, this is not enough to accomplish a successful bowel situation, and therefore, 68% of the patients used enemas regularly. Compared to a Swedish study [8], the children in this study were subjected to more extensive medical treatment.

Due to the risks and patient-related obstacles for surgery, an appendicostomy is often considered the final step in the struggle of achieving satisfying bowel function. This is demonstrated by the fact that only four patients in this study had received an appendicostomy, while there were an additional six patients with inadequate results. Perhaps these children would have experienced improved bowel function with the help of this procedure.

Great individual variation in terms of the quantity and type of surgical procedure was found in the children included in this study. More than half of the patients did not undergo surgery at all, showing that a great number of the procedures were performed on a small group of patients. Of the 26 patients who underwent surgical procedures, 24 patients suffered from myelomeningocele, significantly showing that the children with more extensive defects also were the ones with more congenital malformations, or symptoms, requiring surgical treatment. There may be several reasons why many children did not undergo any surgeries. It is obvious that some of the children included were too young to have surgery for incontinence. It was noted that some urogenital and gastrointestinal investigations and surgeries were postponed due to other issues connected to the spina bifida diagnosis, which gave these patients delayed treatment for their constipation and therefore, inadequate outcomes in this study. This is a continuous problem when investigating children involved in a multidisciplinary follow-up. Another possible explanation as to why we found a low rate of urologic surgery might be local traditions. We found that there was potential to improve the treatment given when evaluating results of patient satisfaction concerning urinary incontinence. It would be of great value to perform a study comparing the results concerning patient satisfaction in regard to urinary incontinence as well as renal function between centers with different approaches in management to investigate if patients are more satisfied in centers with more proactive surgical management of incontinence surgery and if surgery influences renal function.

Due to the large catchment area, a great amount of healthcare personnel from different hospitals were involved in these patients. This affects communication and can result in differences in follow-up time for patients living in the same medical district as the tertiary center compared to those receiving some investigative measures and treatments from another medical district. The national follow-up program, MMCUP, was created in order to counteract these differences, but the program has not yet been implemented by all healthcare employees in Sweden and therefore, has not been used to its full potential.

This study has several limitations besides its retrospective design. Patient-reported outcomes were interpreted from records, and no validated questionnaires were used which would have made it possible to compare the results to previous literature or with healthy controls. Furthermore, this study did not include results from video urodynamics.

Conclusively, a significant portion of the children reported inadequate results regarding their bladder and bowel management, indicating the importance of this study and the need for further healthcare improvement. Surgical procedures in the urogenital and gastrointestinal tract were performed in a defined group of patients.

## Figures and Tables

**Table 1:** Demographics and congenital malformations of 62 patients with spina bifida.

	Patients (*n* = 62)	
Age (months)	125 (11–210)	
Gender (F/M)	32/30	52%/48%
Born in Sweden (Yes/No)	44/18	71%/29%
Diagnosis:		
MC	5	8%
MMC	47	76%
Unknown	10	16%
Congenital malformation (Yes/No)	28/34	45%/55%
Urinary tract malformations *	36	
Gastrointestinal tract malformations *	3	
Abdominal wall malformations *	11	

Values presented as median (min–max) or the absolute number and percentage, %, of patients. * quantity in total. F = female, M = male, MC = meningocele, and MMC = myelomeningocele.

**Table 2:** Bladder management in 62 patients with spina bifida.

	Patients (*n* = 62)	
CIC:		
Age at start of CIC ^a^ (days)	217 (0–4378)	
CIC at latest follow up (Yes/No)	47/15	76%/24%
CIC at some point (Yes/No)	55/7	89%/11%
Anticholinergic treatment:		
Age at start ^b^ (months)	32 (0–149)	
Treatment at latest follow-up ^c^ (Yes/No)	36/24	58%/39%
Treatment at some point (Yes/No)	40/22	65%/35%
Botox (Yes/No)	2/60	3%/97%
Bladder neck injection (Yes/No)	4/58	6%/94%
Bladder neck plasty (Yes/No)	0/62	0%/100%
Bladder augmentation (Yes/No)	2/60	3%/97%
Alternate CIC-channel ^d^ (Yes/No)	2/60	3%/97%
Urinary diversion ^e^ (Yes/No)	3/59	5%/95%
Results		
Satisfying	22	35%
Acceptable	16	26%
Inadequate	23	37%
Unknown	1	2%

Values presented as the absolute number, *n*, and percentage, %, of patients. ^a^ Age at start of CIC within the patient group born in Sweden was 10 (0–1430) days; ^b^ age at start of anticholinergic treatment within the patient group born in Sweden was 25 (4–99) months; ^c^ data regarding anticholinergic treatment was unknown in two patients; ^d^ one Monti and one Mitrofanoff; ^e^ one vesicostomy, one suprapubic catheter, and one unknown. CIC = clean intermittent catheterization.

**Table 3:** The presence of vesicoureteral reflux (VUR), antibiotic treatment, follow-up, and surgery in 55 patients with spina bifida.

	Patients (*n* = 55)	
Age at first VCUG (days) ^a^	39 (5–5093)	
Reflux (Yes/No)	21	38%
Bilateral	4	19%
Grade 1 (Right/left)	1/0	
Grade 2	2/4	
Grade 3	6/4	
Grade 4	2/4	
Grade 5	1/1	
Prophylactic antibiotics ^b^ (Yes/No)	13/2	87%/13%
Reflux at follow-up (Yes/No)	9/4	69%/31% 44%
Bilateral	4	
Grade 1 (Right/left)	0/0	
Grade 2	2/0	
Grade 3	4/2	
Grade 4	1/3	
Grade 5	1/2	
No follow-up	2	
No indication for follow-up	6	
Time to follow-up (months) (*n* = 13)	11 (5–34)	
Deflux (Yes/No)	3/18	14%/86%
Ureteric reimplantation (Yes/No)	1/20	5%/95%

Values presented as the absolute number, *n*, and percentage, %, of patients. ^a^ Age at first VCUG within the patient group born in Sweden was 29 (5–628) days; ^b^ patients who received prophylactic antibiotics due to VUR of grade 3 or higher. VCUG = voiding cystourethrogram, VUR = vesicoureteral reflux.

**Table 4:** Bowel management in 62 patients with spina bifida.

	Patients (*n* = 62)	
Laxatives:		
Yes	45	73%
No	11	18%
Occasionally	6	10%
Enema:		
Yes	39	63%
No	16	26%
Occasionally	7	11%
Appendicostomy (Yes/No)	4/58	6%/94%
Result		
Satisfying	32	52%
Acceptable	22	35%
Inadequate	7	11%
Unknown	1	2%

Values are presented as the absolute number, *n*, and percentage, %, of patients. MACE = Malone antegrade continence enema.

**Table 5:** Surgeries among 62 patients with spina bifida.

Operations	
Total	109
Patients with one or more surgery	26 (42%)
Operations/patient	3 (1–13)
Urogenital surgeries (*n* = 18 patients with 58 operations):	
Cystoscopy	11
Bladder neck injection	8
Orchidofunicolysis	7
Suprapubic catheter	7
Botox	6
Deflux	4
Urinary stoma	4
Bladder augmentation	2
Circumcision	2
Ureteric reimplantation	2
Bladder diverticulum	1
Orchiectomy	1
Pyeloplasty	1
Pyelostomy	1
Ureteral catheter	1
Gastrointestinal surgeries (*n* = 14 patients with 46 operations):	
Inguinal hernia	14
Gastroscopy	6
MACE	4
Closure of gastrostomy	3
Esophageal dilation	3
Esophageal pH-test	3
Gastrostomy	3
Rectopexy	3
Bowel stoma	2
Rectoscopy	2
Enteroscopy	1
Intestinal malrotation	1
Surgery for cloacal malformation	1

Values are presented as the absolute number, *n*, and percentage, %, of patients. MACE = Malone antegrade continence enema.

## Data Availability

Data supporting reported results can be received upon request to the authors.
